# Innovative financing for HIV response in sub–Saharan Africa

**DOI:** 10.7189/jogh.06.010407

**Published:** 2016-06

**Authors:** Rifat Atun, Sachin Silva, Mthuli Ncube, Anna Vassall

**Affiliations:** 1 Harvard T.H. Chan School of Public Health, Harvard University, Boston, USA; 2 Health Policy Programme, Imperial College London, London, UK; 3 Blavatnik School of Government, Oxford University, Oxford, UK; 4 London School of Hygiene and Tropical Medicine, London, UK

## Abstract

**Background:**

In 2015 around 15 million people living with HIV were receiving antiretroviral treatment (ART) in sub–Saharan Africa. Sustained provision of ART, though both prudent and necessary, creates substantial long–term fiscal obligations for countries affected by HIV/AIDS. As donor assistance for health remains constrained, novel financing mechanisms are needed to augment funding domestic sources. We explore how Innovative Financing has been used to co–finance domestic HIV/AIDS responses. Based on analysis of non–health sectors, we identify innovative financing instruments that could be used in the HIV response.

**Methods:**

We undertook a systematic review to identify innovative financing instruments used for (1) domestic HIV/AIDS financing in sub–Saharan Africa (2) international health financing and (3) financing in non–health sectors. We analyzed peer–reviewed and grey literature published between 2002 and 2014. We examined the nature and volume of funds mobilized with innovative financing, then in consultation with leading experts, identified instruments that held potential for financing the HIV response.

**Results:**

Our analysis revealed three innovative financing instruments in use: Zimbabwe’s AIDS Trust Fund (a tax/levy–based instrument), Botswana’s National HIV/AIDS Prevention Support (BNAPS) International Bank for Reconstruction and Development (IBRD) Buy–Down (a debt conversion instrument), and Côte d'Ivoire's Debt2Health Debt Swap Agreement (a debt conversion instrument). Zimbabwe’s AIDS Trust Fund generated US$ 52.7 million between 2008 and 2011, Botswana’s IBRD Buy–Down generated US$ 20 million, and Côte d’Ivoire’s Debt2Health Debt Swap Agreement generated US$ 27 million, at least half of which was to be invested in HIV/AIDS programs. Four additional categories of innovative financing instruments met our criteria for future use: (1) remittances and diaspora bonds (2) social and development impact bonds (3) sovereign wealth funds (4) risk and credit guarantees.

**Conclusion:**

A limited number of innovative financing instruments contributed a very modest share of funding toward domestic HIV/AIDS programs. Several innovative financing instruments successfully applied in other sectors could be used to augment domestic financing toward HIV/AIDS programmes.

By 2015, around 15 million individuals were accessing life–saving antiretroviral treatment (ART) [1]. Yet, the “AIDS transition” [2] is not in sight–in 2014, there were 36.9 million people living with HIV, 2 million new HIV infections and 1.2 million AIDS–related deaths [3].

The continuing HIV epidemic requires sustained investment to prevent new infections and to provide treatment to those who need ART now and in the future. However, as more individuals access ART, domestic obligations for financing HIV will increase, reaching an estimated US$ 190 billion between 2015 and 2050 and account for as high as 47% of the GDP in high prevalence sub–Saharan African countries such as Malawi [4], creating long term commitments that have to be met. The financial obligations have major implications for the affected countries and donors–not just in economic terms, but also in the way they manage financing of the HIVresponse, which requires empowering countries to take greater responsibility for managing funds from all sources.

The return of investment in HIV response as measured by benefit to cost ratio has been estimated at 280% [5], with substantial economic, social and health benefits reported by other studies [6], comparable to benefit to cost ratios reported for maternal and child health investments [7]. However, donor financing, which accounts for a large share of the HIV/AIDS funding in sub–Saharan Africa, is constrained due to the global economic crisis [8]. Domestic financing remains equally challenging, especially in high–prevalence low–income countries that are fiscally constrained. Compounding the financing challenges are inefficiencies in channeling and use of available funds, and the harmful asymmetry between the long–term financing needs for HIV and short–term replenishment cycles of donor institutions [9].

There are opportunities for increasing HIV financing, however. African economies are enjoying economic growth [10]. There are also untapped natural resources that could generate upwards of US$ 4 billion each year [11]. Additional fiscal space could be created in domestic budgets by improving efficiency in allocation both to and within HIV programmes and by co–financing HIV services with funding other economic sectors [12]. Innovative financing could offer new sources of funding. Conceived as a funding source to meet the Millennium Development Goals (MDGs), innovative financing, which provided around US$ 6 billion in total in 2002–2012 [13], is increasingly an important source of funding for global health [14].

We explore how innovative financing could be used to co–finance the long–term HIV obligations by augmenting domestic contributions in sub–Saharan Africa. We analyze how different innovative financing instruments can be operationalised and the institutional arrangements needed for their effective use.

## METHODS

We undertook a systematic review to identify domestic innovative financing used to augment funding for HIV programmes in sub–Saharan African countries. We then extended the search to identify innovative financing used in global health and non–health sectors.

We searched peer–reviewed literature and grey literature published between 2002 and 2014, the period when innovative financing gained prominence following the United Nations International Conference on Financing for Development [15], and the publication of the High Level Taskforce on Innovative International Financing for Health Systems [16]. We also searched web sites of development agencies, and international financing institutions for published reports and to gather data. We provide the framework for the search, a listing of the data sources and the search strings (Figure S1 in **Online Supplementary Document(Online Supplementary Document)**). In qualifying a financing scheme as innovative, we that took into account the nature of the financing, institutional arrangements and the mode of financing (pooling, channeling and allocation of funds) [13].

We used predetermined criteria (Figure S2 in **Online Supplementary Document(Online Supplementary Document)**) to systematically examine the nature and magnitude of funding that could be mobilised, the characteristics of the innovative financing and their suitability for long–term funding of HIV obligations. We sought views of leading experts involved in innovative financing and development finance (from the World Bank, International Monetary Fund, The African Development Fund, the Global to Fight AIDS, Tuberculosis and Malaria, and the Bill and Melinda Gates Foundation) and in domestic funding of HIV programmes in sub–Saharan countries (such as from Kenya, South Africa, and Ethiopia). We then categorised the innovative financing instruments with the most potential for raising new funds.

We did not consider funding generated from borrowing, funding from health insurance, reprioritisation of existing budgets and efficiency gains from improvements in health systems [17,18], as these approaches are used routinely in funding and managing health budgets.

We analyzed all search results using the Preferred Items for Systematic Reviews and Meta–Analysis (PRISMA) guidelines [19]. We present all monetary amounts in 2010 US Dollar (US$) equivalents based on World Bank official exchange rates [20] and the GDP deflator indices from the US Department of Commerce, Bureau of Economic Analysis, National Income Product Accounts Tables [21]. We report our findings by calendar year.

## RESULTS

### Innovative financing from taxes, levies and debt conversion instruments

The systematic review revealed three innovative financing instruments using national taxes and levies and debt conversion for countries including debt buy–down currently in use for HIV/AIDS, namely: Zimbabwe’s AIDS Trust Fund [22], Botswana’s National HIV/AIDS Prevention Support (BNAPS) International Bank for Reconstruction and Development (IBRD) Buy–Down [23], and Côte d'Ivoire's Debt2Health Debt Swap Agreement [24].

We briefly mention three other innovative financing instruments which were intended but were not established as functioning entities, were established and then terminated, or had failed to generate meaningful revenues.

### Taxes and levies

Established in 2000, Zimbabwe’s AIDS Trust Fund received proceeds from a 3% tax levied on formal sector employers and employees. Of the total funds collected, 50% was earmarked for ART programmes, 10% for prevention programmes, and the remainder toward program administration and support. The funding generated in 2000–2008 was not available as the estimated figures were distorted due to hyperinflation. In 2008–2011, the levy generated US$ 52.7 million (89.8% of the US$ 58.7 million in total domestic public HIV spending [25]), with US$ 5.7 million generated in 2009, US$ 20.5 million in 2010 and US$ 26.5 million in 2011 [26].

We identified other tax/levy–based instruments, which have been proposed, but not fully scaled up or implemented. For example, starting in fiscal year 2015/16, Kenya is establishing an HIV investment unit that will develop a model for resourcing a new HIV Trust Fund which will be created within the National AIDS Control Council, and will seek to mobilise resources from domestic and international sources, including matching funds, corporate social investments, debt swaps, infrastructure bonds and the informal sector [27], but the scheme is not yet operational.

The government of Uganda has proposed to establish a HIV Trust Fund (based on the Zimbabwe model) to provide sustainable financing for HIV programmes. The HIV Prevention and Control Act, 2014 has stipulated the source of income for the Fund from levies (2% of the total tax revenues) on beers, spirits, soft drinks and bottled water, in addition to income from international sources [28].

Tanzania has also established an AIDS Trust Fund in 2015 through the Tanzania Commission for Aids (Amendment) Act 2014 enacted by the Parliament in early 2015 to reduce donor dependence [29].

Trust Funds are a new and a promising new approach that pools funds revenues from multiple sources [30]. Here the innovation is less about the source of financing but more about the pooling and application of funds [13].

Tax and levy–based instruments have the potential increase in revenues in sub–Saharan Africa, (where trade taxes have declined since 1990s and income taxes have stagnated since 2000 [31]) and where revenue streams from extractive industries that could be taxed [11]. Revenues from taxes and levies can be earmarked or ring–fenced for HIV programs within public finance budget (as with Zimbabwe’s AIDS Levy). However, ring–fencing or earmarking reduces fungibility of public funds [32] and may limit a government’s ability to respond to unexpected shocks and to adjust allocations for short–term priorities [33].

### Debt conversion

Buy–downs convert credits to grants, often with conditions [34]. Debt conversion instruments can be operationalised using a combination of schemes. With bilateral conversion, where the lender simply cancels all or a portion of the loan or credit, operationalisation means ensuring that the conditions for cancellation/forgiveness are met. In most instances, program monitoring is mediated via the respective ministries and debt cancellation occurs ex–post [34]. With trilateral conversion, if the lender cancels or forgives all or part of a loan with the expectation that the debtor invest that portion in a multilateral institution, a modified debt arrangement maybe required. If on the other hand, a third party donor purchases all or part of a loan either conditionally or unconditionally, the execution of the debt arrangement must occur ex–ante due to the inclusion of additional institutions [34].

Botswana’s National HIV/AIDS Prevention Support IBRD Buy–Down of US$ 50 million was used to address implementation gaps in the domestic HIV response. The program also supported the implementation of a new national operational plan for scaling up prevention as a national “survival strategy”. A buy–down of US$ 20 million, supported by the European Commission was later introduced in Botswana to reduce HIV prevalence in young adults with conversion predicated on the HIV program meeting performance objectives [35].

A similar but more recent buy–down under Debt2Health, a novel debt conversion instrument managed by the Global Fund [36], provided US$ 27 million to the domestic HIV response in Côte d'Ivoire. In exchange for the creditor (Germany) forgoing the US$ 27 million debt, Côte d'Ivoire was required to invest at least half of the proceeds on national HIV treatment and prevention programmes [37]. While Debt2Health has financed several HIV/AIDS programmes worldwide, Côte d'Ivoire is the only African country to benefit from the instrument.

Between 2001 and 2011, sub–Saharan Africa received approximately US$ 2 billion in concessional aid for HIV/AIDS programmes [38], which offers potential for the use of Debt2Health. Between 2011 and 2013, debt conversion programmes totaling US $45.7 million were signed using Debt2Health [39], including US$ 27 million for Côte d'Ivoire.

We summarize in **Table 1** the key features of the innovative financing instruments discussed.

**Table 1:** Innovative financing instruments in use in sub–Saharan Africa, those planned, and their features

Instrument name	Operational status	Year established	Instrument type	Source of revenue	Financing agent	Revenues generated/forecast
Botswana’s National HIV/AIDS Prevention Support (BNAPS) IBRD Buy–Down	Operational	2009	Debt buy–down	Concessional or non–concessional debt	European Commission (up to US$ 20 million)	US$ 50 million (over five years)
Côte d'Ivoire's Debt2Health Debt Swap Agreement	Operational	2010	Debt swap	Concessional or non–concessional debt	Government of Germany	US$ 27 million
Zimbabwe’s AIDS Trust Fund	Operational	2000	Levy	Formal sector employee and employer income	Domestic government	US$ 85.2 million (through 2012)
Kenya's National Aids Control Council (NACC) Tax	Not yet operational	Not yet established	Multiple sources	Multiple sources	Domestic government, augmented with funds from external sources	Unknown
Uganda's HIV Trust Fund	Not yet operational	Established by HIV and AIDS Prevention and Control Act, 2014	Tax	Tax revenue from alcohol, soft drinks and bottled water	Domestic government and international sources	Unknown
Tanzania's AIDS Trust Fund	Not yet operational	Established in 2015	Not specified	Not specified	Domestic government	Unknown

### Airline levy and contributions from retail sales

In addition to innovative financing from taxes and levies and debt conversion international innovative financing such as Airline Solidarity Levy (Airline Levy) [40] and PRODUCT(RED)^TM^ [41] have been used to generate financing for HIV/AIDS programmes. Airline Solidarity Levy is domestically implemented with revenues pooled and channeled via UNITAID. In Africa, distinct from the Airline Solidarity Levy, several countries have introduced their own versions of airline levies, including Cameroon, Congo, Madagascar, Mali, Mauritius and Niger, and is under consideration in Benin, Burkina Faso, Central African Republic, Gabon, Guinea, Kenya, Liberia, Namibia, Senegal, São Tomé and Principe, and Togo [40]. Product(RED) generates revenue through direct contributions via retail sales in Western countries the proceeds of which are then channelled to the Global Fund to Fight AIDS, Tuberculosis and Malaria [42].

### Innovative financing for other services with potential future use for HIV/AIDS

Our analysis of global health and non–health sectors revealed four additional categories of innovative financing with the potential to expand fiscal space and provide additional funding for HIV/AIDS, namely: remittances and diaspora bonds; social and development impact bonds; sovereign wealth funds; and guarantees (See Panel in **Online Supplementary Document(Online Supplementary Document)** for a summary of these categories).

### Remittances and diaspora bonds

In 2014, remittances accounted for US$ 67.1 billion of the US$ 206.6 billion in external flows to sub–Saharan Africa. Nigeria was the largest recipient with US$ 21 billion in 2013 [31]. Remittances are additional and particularly attractive given their stability in comparison to direct foreign investment or private financing flows.

Remittances can be mobilised via issuance of diaspora bonds, which have been successfully used in India (since 1991), in Israel (since 1951) and in Sri Lanka (since 2001). A diaspora bond can be developed either directly by a government or a state–owned bank. The bond can be setup to raise revenues on a continuous basis (annual issuance) or on an on–demand basis (opportunistic issuance), and can be established as non–negotiable fixed rate or as floating rate bonds or notes in different denominations. Fixed rate bonds, which are inherently less volatile, provide increased predictability to financing [43].

Diaspora bonds offer several benefits to issuers. Due to investors’ “patriotic motivations”, the issuer could conceivably offer a lower rate of return, thereby gaining a “patriotic discount”. The bonds also offer the issuer an opportunity to improve sovereign credit rating by creating a new funding source. For the investor, aside from meeting personal motivations, the bonds offer the flexibility to receive interest and principal in issuer currency, which can be routed to meet liabilities in the issuing country [43].

While revenues from India’s diaspora bonds were used to offset the country’s balance of payment crisis in 1991 [44], the Israeli diaspora bonds have financed public works such as seawater desalination, housing construction and communication infrastructure [43]. Though we did not uncover evidence of the use in health programs, the characteristics of these instruments suggest that it could be a viable source.

### Social Impact Bonds and Development Impact Bonds

Impact bonds have gained prominence as a means to attract and “crowd in” additional private capital to address social challenges. Social Impact Bonds and Development Impact Bonds allow private investors to invest in social causes and generate suitable financial returns, contingent on the quality of the outcomes achieved [45]. In a Social Impact Bond the outcome payer is the government, while in a Development Impact Bond the outcome payer is a donor, development agency or a philanthropic foundation [42]. In incentivising payment based on the quality of the outcomes achieved, the Development Impact Bonds offer the potential to maximise impact underpinned by rigorous monitoring and evaluation. For the sponsoring government or agency (the bond issuer), Development Impact Bonds offer risk protection and potential overall cost savings in implementing programmes.

When implementing a social or a development impact bond however, governments typically focus on social programmes with proven interventions, which fall within investor risk thresholds, can generate cost savings, have well–defined target populations and have quantifiable impacts/outcomes. Thus in the health sector, social or development impact bonds are most appropriate for preventative rather than treatment interventions, as the cost of the latter should be met from operational budgets, and borrowing through bonds should be used to invest and not fund operational expenditures. The ethics of targeting interventions with easy to measure outcomes rather than those with the potential to meet most need is debatable, however.

While social impact bonds have proven successful in non–health sectors, including recidivism reduction in the United Kingdom [46], education and housing in the United States [47], they are yet to be implemented in for HIV prevention and control. Several case studies indicate the potential for the health sector however, including in implementing Treatment as Prevention programmes and tuberculosis control programmes in Swaziland [48] and malaria control programmes in Mozambique [49].

### Sovereign Wealth Funds

Sovereign Wealth Funds (SWFs) are special purpose investment funds owned by governments, which are established for creating stable returns on the funds invested for macroeconomic stability, to meet contingent liabilities, and to withstand economic shocks [50]. In 2012, total assets of SWFs accounted for approximately US$ 3 trillion. Based on IMF and the Santiago Principles taxonomy [51], four types of SWFs are distinguishable, namely: stabilization funds, savings funds, development funds (reserve investment fund) and pension reserve funds. Stabilization funds absorb macroeconomic shocks due to commodity price volatility and other external events (eg, Chile’s Economic and Social Stabilization Fund). Savings funds preserve wealth for future generations by transforming nonrenewable resources into monetary assets (eg, the Abu Dhabi Investment Authority). Development funds aim to finance social development and infrastructure (eg, Mubadala in United Arab Emirates) [52], whereas pension reserve funds aim to fund pension–related contingent–type liabilities (eg, Norway’s Government Pension Fund).

African Sovereign Wealth Funds accounted for US$ 114.3 billion in 2009, approximately 3% of global sovereign wealth funds at the time. By 2012 there were 15 Sovereign Wealth Funds in Africa with the four largest sourced from oil and gas revenues and the fifth sourced from diamonds, minerals and other natural resources, but amounts used as development funds are unknown, due to scarcity of publicly available data [53].

### Guarantees

Guarantees can be used to catalyze private financing by mitigating risks, especially those that are political, contractual or regulatory in nature. The largest volume of guarantees originated from the World Bank Group, which provided by 2013 US$ 4.5 billion as 37 guarantees across 30 countries [54]. The guarantees were sourced from International Development Association (IDA), IBRD, International Finance Corporation (IFC) [55] or the Multilateral Investment and Guarantee Agency (MIGA) [56], and structured as Partial Risk Guarantees, Partial Credit Guarantees or Policy Based Guarantees.

Partial Risk Guarantees support private sector investment including in public–private partnerships. Partial Credit Guarantees support commercial borrowing in support of public investment projects and Policy Based Guarantees support commercial borrowing for budget financing or reform programmes. Partial Risk Guarantees are available to all IBRD and IDA countries, while Partial Credit Guarantees and Policy Based Guarantees are only available to IBRD–eligible countries [54]. Aside from the World Bank and other multilateral development banks, private foundations such as the Bill & Melinda Gates Foundation also provide guarantees [57].

Guarantees offer several benefits to borrowers. The reduction of default risk improves potential of the country for securing loans and thereby stimulates additional investment. Guarantees can also reduce the cost of capital due to lower interest rates afforded to the borrowing government via guarantor’s credit worthiness (especially in the case of the World Bank due to the bank’s AAA rating) [54]. Guarantees allow governments to share the risk of projects with the private sector. In the case of World Bank guarantees, capacity building is also afforded as an added benefit [58]. While guarantees are beneficial to borrowers they create risks to guaranteeing entities, which with the prevailing global economic crisis may limit the potential for expanding guarantees.

Between 2005 and 2012, the IBRD mobilised US$ 1.2 billion and IDA mobilised US$ 789 million as guarantees. The energy sector received the highest volume of financing (US$ 2 billion) with IFC or MIGA guarantees, with the largest proportion within the African region (US$ 1.1 billion). The health sector however, has yet to receive guarantees from the World Bank Group [54]. By contrast, the Gates Foundation has issued credit enhancement guarantees to enhance affordability of vaccines and health commodities amounting to around US$ 250 000, US$ 500 000 and US$ 400 000 in gross exposure in 2012, 2013 and 2014 respectively [56].

Borrowing increases future financial liabilities for countries, but by reducing the cost of borrowing, guarantees can significantly reduce the cost of servicing the debt burden. The benefits of guarantees can be further augmented if countries demonstrate economic returns from HIV treatment/prevention programmes via reduced HIV incidence and enhanced labour productivity that benefit the economy.

## DISCUSSION

We identified limited use of innovative financing instruments in domestic financing of HIV programmes in sub–Saharan Africa. The findings suggest both an opportunity to augment domestic financing, but also a possible hindrance of innovative financing due to weak domestic political or regulatory climates – only three innovative financing instruments were in current use and operationalised to generate meaningful revenues.

The instruments that were successfully implemented were either based on debt conversion or taxes and levies, however with no new instruments that unlocked resources to generate additional predictable revenues from new sources beyond taxes/levies and debt. Innovative financing included both new financing instruments, but also innovative ways of using existing instruments for HIV–for example debt conversion and levies.

The revenues generated through tax/levy mechanisms (US$ 52.7 million in Zimbabwe), accounted for a relatively minor share of the domestic HIV budget [26]. The revenues generated from debt conversion were also modest (US$ 50 million in Botswana [23] and US$ 27 million in Côte d'Ivoire [24]). Similarly, despite the growth of bond issuance by countries of sub–Saharan Africa [10], there are no bond–based instruments for HIV, even though bonds have been used to finance malaria programmes in Mozambique [59].

While the relative absence of innovative financing instruments that generate new sources is a challenge, the nascent space for innovative financing is also an opportunity as countries affected by HIV move to increase domestic share of the financing obligations for HIV. The Global Fund’s country graduation and counterpart financing [60] and PEPFAR’s multi–year partnership framework agreements [61] will encourage this transition. Recent evidence suggests that 12 high prevalence sub–Saharan African countries would be able to finance as much as 64% of future financing needs in 2014–2018 through economic growth [9]. Much of the additional financing in these countries would be from the expansion of domestic fiscal space from traditional sources [40], which could be further augmented by innovative financing, if constraints for introducing new funding instruments are overcome. In this context, innovative financing instruments that enable securitisation of future income streams offer the most immediate possibility of augmenting existing funding to accelerate the HIV response, though the risk of exaggerating already high future debt obligations has to be carefully considered. Securitisation is an area where guarantees could be leveraged, as guarantees enable the borrowing countries to substantially reduce the interest payable on the debt while reducing default risk to lenders–thereby unlocking new funds from private sector investors by making the benefit–risk calculus more attractive.

Taxes and levies continue to be promising sources of new revenue if implemented as a modest charge on high volume transactions (especially on goods such as tobacco and alcohol with well–proven health harm) to expand the government revenue base with revenues pooled and effectively committed. Diaspora bonds are hitherto untapped sources of funding for the HIV response, due to their long–term nature, which is aligned with the long–term obligations for HIV. However, apart from the diaspora bonds issued in India and Israel, their widespread uptake is limited [43].

Sovereign wealth funds offer potential new funding sources if they could be used to leverage private sector investments, through public–private partnerships to invest in health system infrastructure or in new ventures to create additional capacity for health service provision in the health system, where new providers could be contracted by the government to provide services.

Social impact bonds offer significant potential for mobilizing new and additional external resources from private sources. Social impact bonds would be particularly suitable for financing preventative interventions that reduce future burden of disease, especially for effective interventions that are under–utilized or inefficiently delivered (such as prevention of mother to child transmission, harm reduction, voluntary male circumcision, condom distribution and use) to bring health, economic and social benefits and achieve returns beyond the costs. By transferring the risk of success to private investors that bring new funds and innovative service delivery models to the sector, the government (or the public sector funding entity) pays for successful outcomes. The investors, be it philanthropic foundations, high net worth individuals or socially conscious funders, receive financial return and social benefits.

To maximise the benefits of revenues generated from domestic innovative financing, the “partnership” arrangements between donors and HIV–affected countries must be reframed. In spite of the Paris Declaration on Aid Effectiveness and the Accra Agenda for Action [62], country ownership of donor funding and health programmes has yet to be achieved in most settings. Instead, the partnership remains disproportionately weighted toward donors, especially in countries where donor financing outweighs domestic finding. For example, in Uganda domestic financing for HIV accounted for US$ 53 million in 2013, whereas donor financing accounted for US$ 446 million [9]. In Malawi, the donor share of HIV funding was 98% of cumulative spend [63], although the appropriate balance of domestic and international financing is debated [64].

In restructuring the partnership, domestic governments’ view that a duty lies with the donor must be balanced with a view that emphasizes mutual responsibility and accountability to ensure sustainable and predictable financing for HIV response. In low income countries with high prevalence of HIV, as HIV financing accounts for a large portion of the health budget and the GDP [9], however, long term financing consideration need to rest with the ministry of finance and the government in general, which have the responsibility for management of debt levels and priorities in the available fiscal space. Similarly, donor perceptions that shifting resources among recipient countries is acceptable, as long as outlay commitments are met, should also be overcome.

“Commitment Technologies”, which can be utilized to enforce binding and credible commitments, both from donors and recipients, can capitalise on several key features of the status quo arrangements. Presently, donors reap the most benefit from efficiency gains in development assistance. Recipients on the other hand, are uncertain of benefits, as the returns are intangible in budgetary terms, although health benefits are real. If donors (or investors) make multi–year commitments to invest in a proportion of need, they stand to reap a share of efficiency improvements equal to participation. Similarly, if the incentives for domestic governments can be made more tangible, via the use of mechanisms such as social impact bonds^46^, results–based financing [65] or cash–on–delivery [66] for example, where results to be delivered equate to reduction in incidence, then the prospects for reframing the partnership become promising.

The opportunity for innovative financing to augment domestic financing for HIV/AIDS is real and important given the magnitude of the long–term obligations in sub–Saharan Africa estimated at around US$ 180 billion [4]. Sustaining HIV response in the era of sustainable development with competing priorities makes the search for funding from innovative financing all the more pressing.
