# Supplementary material for: Innovative financing for HIV response in sub–Saharan Africa
Source: J Glob Health. 2016 Feb 28;6(1):010407. doi: 10.7189/jogh.06.010407 (PMC4871060; doi:10.7189/jogh.06.010407)
Supplement: Online Supplementary Document [file jogh-06-010407-s001.pdf]

Online Supplementary Document

Atun et al. Innovative financing for HIV response in sub–Saharan Africa

JoGH 2016;6:010407

Figure S1. PRISMA^{TM} Diagram for Systematic Review.

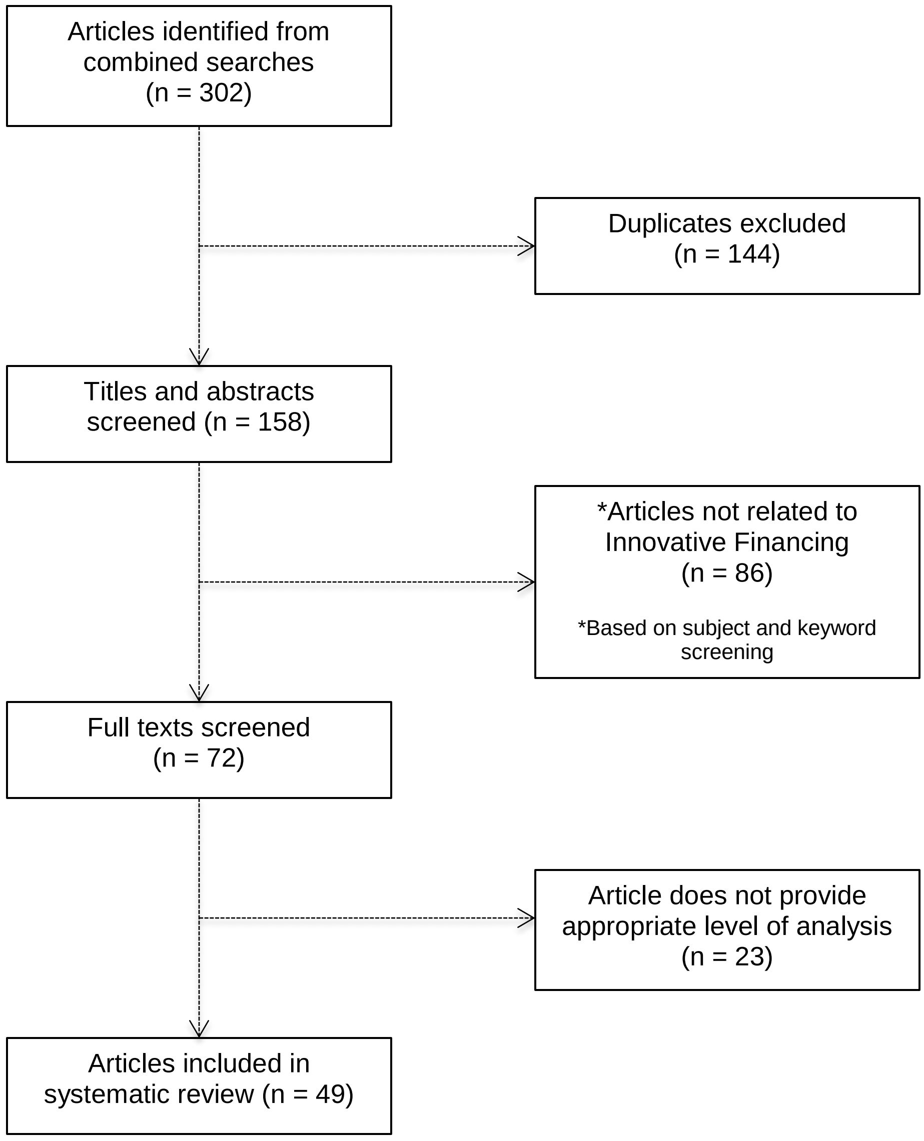

Figure S2. Instrument selection scheme.

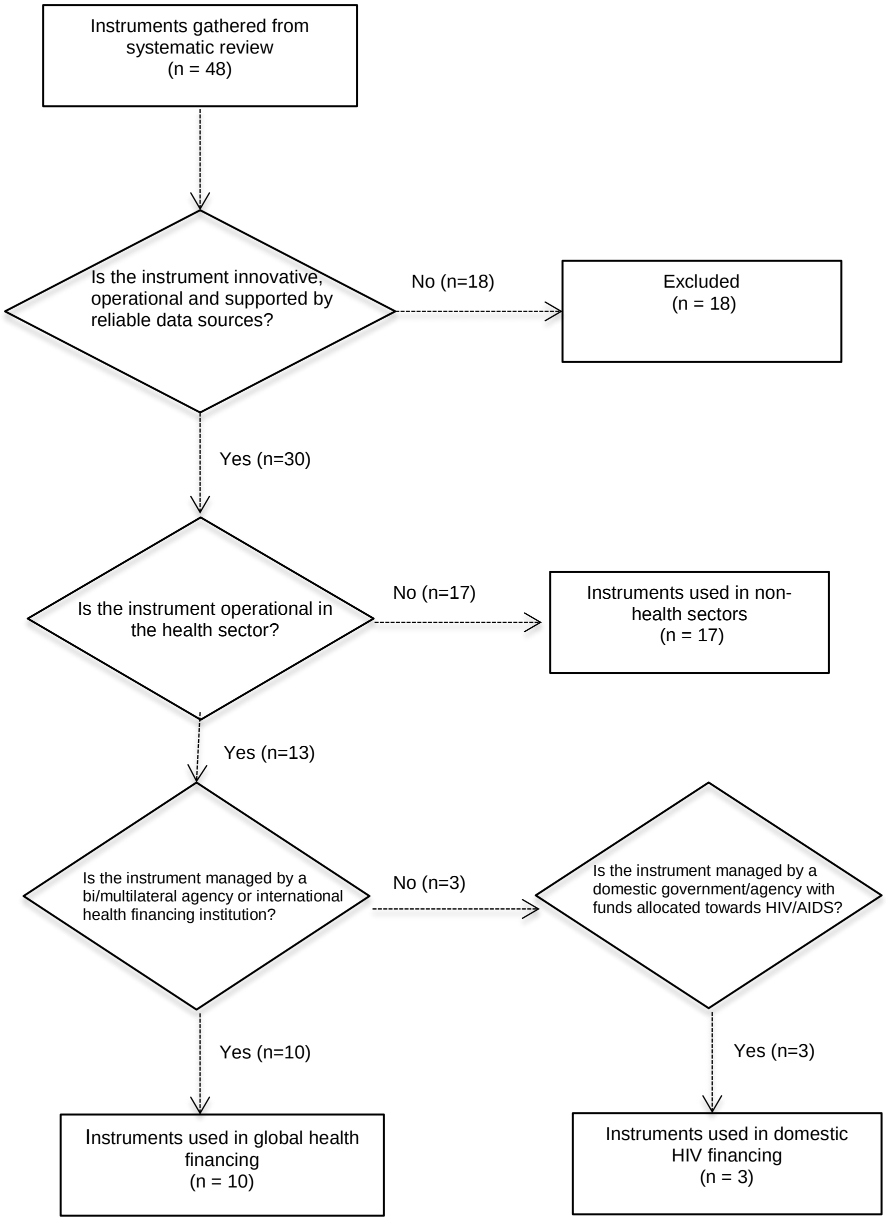

Panel. Innovative Financing Instruments used in health, environment and education sectors

1. Debt Conversion Instruments

Debt conversion as a means of financing social investment gained popularity following the launch of the Heavily Indebted Poor Countries (HIPC) Initiative in 1996 that linked debt relief to poverty reduction[1]. Since then a range of debt conversion instruments have proliferated within various sectors. The most prominent have been debt buy-downs, based on lending by the World Bank Group, either as non-concessional IBRD[2] or concessional IDA[3] and lending by the Islamic Development Bank[4].

Buy-downs allow a third party to buy-down all or a part of a loan, either by softening the loan terms or by paying down interest and/or principal amount. The buy-down releases the loan recipient from all or part of future repayment obligation, thereby allowing those resources to be earmarked for social programs. The strength in buy-down arrangements stems from the third party mandating that the borrower invest the repayment resources in social programs. Often buy-down terms are tied to performance objectives, further enhancing efficacy. On one hand, buy-downs entice countries to borrow; on the other they create an incentive for programs to focus on performance in order to qualify and gain concessionality. The buy-down also allows front-loading of the repayment to the lender thereby making those funds recyclable for future borrowers.

2. Social Impact Bonds

Social impact bonds (SIBs) are instruments used to fund welfare and social sector investments by bringing together government, service providers and external investors.[5] Social impact bonds have been used as innovative financing to attract private capital to help achieve social outcomes[6]. SIBs originated in the UK in 2010, with the first such investment targeted at reducing recidivism among select prison populations, and have since expanded

---

[1] Imf.org, (2015). Factsheet -- Debt Relief Under the Heavily Indebted Poor Countries (HIPC) Initiative. [online] Available at: https://www.imf.org/external/np/exr/facts/hipc.htm [Accessed 1 Feb. 2015].

[2] Web.worldbank.org, (2015). IBRD. [online] Available at: http://web.worldbank.org/WBSITE/EXTERNAL/EXTABOUTUS/EXTIBRD/0,,menuPK:3046081~pagePK:64168427~piPK:64168435~theSitePK:3046012,00.html [Accessed 3 Feb. 2015].

[3] Worldbank.org, (2015). IDA Financing. [online] Available at: http://www.worldbank.org/ida/financing.html [Accessed 3 Feb. 2015].

[4] Isdb.org, (2015). Islamic Development Bank. [online] Available at: http://www.isdb.org/irj/portal/anonymous?guest_user=idb_en [Accessed 1 Feb. 2015].

[5] Cabinet Office. Centre for Social impact Bonds. https://data.gov.uk/sib_knowledge_box/sib-definition [Accessed 31 October 2015]

[6] Shah, S. and K. Costa, Social Finance: A Primer. 2013, Center for American Progress.

to other sectors, such as homelessness, health, and family support services, in both the UK and US.[7]

A SIB is constructed by a government agency (or a commissioner) that identifies a desired social outcome. An external organization or contractor (the delivery agency) is then engaged to achieve the outcome. A third-party investor (separate from the delivery agency) provides upfront working capital as an at-risk investment. With working capital, the external organization (delivery agency) then sets up programs or interventions to achieve the specified outcome, either through direct service provision or by engaging intermediary service providers. If the desired social outcome is achieved, the government or the commissioner releases payment to the external organization, based on terms specified in an upfront contract. Levels of payment are typically based on the amount of savings that accrue to the government or the commissioner due to the success of the program (e.g., reduced cost of housing prisoners due to lower recidivism rates). If the outcome is not met, the government or the commissioner disburses no payment. The external organization then repays its investors their principal, plus a return on the investment.[5]

3. Development Impact Bonds

Development impact bonds (DIBs) present a variation on the social impact bond structure[8]. The main distinction from a SIB is that with a DIB, payment to third-party implementers upon successful achievement of pre-specified outcomes comes from an external funder (e.g., development agency or charitable foundation), rather than a government or a commissioner. The structure of SIBs and DIBs provide several distinct advantages[9]. First, it makes social problems 'investable' such that private investors can generate a return by paying for interventions to address them. This structure also transfers financial risk from governments to private investors. Depending on the terms of the contract, a SIB with successful outcomes is typically designed to generate savings for the government or the commissioner, as payment for successful service implementation should cost less than the amount the government would have to pay had the new model of service delivery not been implemented.

Success of SIB structuring depends on proven intervention for the given social problem, the intervention aims to achieve a preventative (rather than remedial) outcome which generates savings for the government, the intervention's outcome has an impact on a well-

---

[7] Social Finance (2010). Towards a new Social Economy. Blended value creation through Social Impact Bonds. http://www.socialfinance.org.uk/wp-content/uploads/2014/07/Towards-A-New-Social-Economy-web.pdf [Accessed 31 October 2015]

[8] Center for Global Development and Social Finance, Investing in Social Outcomes: Development Impact Bonds. http://www.cgdev.org/sites/default/files/investing-in-social-outcomes-development-impact-bonds.pdf [Accessed 31 October 2015]

[9] Costa K, Shah S, Ungar S, and the Social Impact Bonds Working Group. Frequently Asked Questions: Social Impact Bonds. 2012. https://www.americanprogress.org/wp-content/uploads/2012/12/FAQSocialImpactBonds-1.pdf [Accessed 31 October 2015]

defined population, raising enough funding for the programme will be challenging through conventional financing mechanisms.[10]

4. Diaspora Bonds

A diaspora bond can be issued by a sovereign entity, sub-sovereign entity or a private corporation to mobilize financing from the diaspora community. Diaspora bond are long-term securities that can be redeemed upon maturity and can provide the issuing country a substantial capital flow.

Patriotism is the primary motivator for investors purchasing diaspora bonds and offers the opportunity to invest in potentially familiar grounds. A recent hypothesis put fourth argues that an incentive for investment in diaspora bonds is the superior access to information and the ability to influence domestic policy. The bonds also offer an opportunity to the diaspora community to minimize risk and diversify their assets away from their countries of residence.

There are an estimated 215 million migrants who live in countries other than their countries of birth; this accounts for more than three percent of the world's population. In 2012, this diaspora transferred an estimate of US$529 billion to their countries of origin, through officially recorded remittances; developing countries received over US$401 billion, a figure projected to grow to US$515 by 2015.

Although a bulk of diaspora investments are in portfolio investments of short-term nature, diaspora resources through diaspora bonds (and remittance backed bonds) represent a key source of funding for development. If one in every ten member of the diaspora community could be persuaded to invest US$1000 in their country, developing countries could raise an estimate of US$20 billion annually for development financing.

5. Sovereign Wealth Funds

Sovereign wealth funds have gained prominence since 2000s with rapid growth in their number and size. They are distinct from public pension funds or reserve assets, and are invested typically in foreign assets in order to provide regular revenues and to protect against macroeconomic shocks.[11] Sovereign wealth funds also invest domestically to secure stable returns that may not be available in international markets.[12]

---

[10] Shah S. Costa K. Social Finance: A Primer. Understanding Innovation Funds, Impact Bonds, and Impact Investing. Center for American Progress. 2013. https://www.americanprogress.org/issues/economy/report/2013/11/05/78792/social-finance-a-primer/ [Accessed 31 October 2015].

[11] www.centralbanking.com, (2015). Who holds the wealth of nations?. [online] Available at: http://www.centralbanking.com/central-banking-journal/feature/2072255/holds-wealth-nations [Accessed 10 Feb. 2015].

[12] Center For Global Development, (2015). Should Sovereign Wealth Funds Finance Domestic Investment?. [online] Available at: http://www.cgdev.org/blog/should-sovereign-wealth-funds-finance-domestic-investment [Accessed 10 Feb. 2015].

The assets of sovereign wealth funds come from national budget surpluses or revenues from extractive industries (such as mining, petrol and gas). The assets are invested to provide a steady income stream (in this sense securitised) to safeguard against economic volatility, reduce liquidity in domestic markets to prevent inflation, and to establish future savings for the current and the next generation.

In 2005, sovereign wealth funds included approximately US$895 billion in cumulative assets, including the United Arab Emirates (US$250 billion), Norway (US$170 billion) and Singapore (US$100 billion). By 2012, sovereign wealth funds accounted for US$7,109.6 billion, with the largest funds held by Norway (US$893 billion), United Arab Emirates (US$773 billion) and Saudi Arabia (US$757.2 billion)[13]. African SWFs had US$114.27 billion in assets under management as of December 2009. Although much less than those in the Middle East and Asia, African SWFs increased from 2008-09, in spite of decreasing oil prices.[13]

The rate of growth, along with several unique characteristics, makes sovereign wealth funds particularly attractive as sources of financing for social programs. Unlike traditional reserves, sovereign wealth funds are more diversified with a broader range of assets with different term, yield and risk profiles. By virtue of having broadly diversified portfolios, sovereign wealth funds are more risk-tolerant than traditional reserves. The risk tolerance allows governments greater flexibility and choice to construct efficient portfolios with a desired return:risk calculus. The returns from sovereign wealth funds have been a valuable source of financing for domestic social investments.

[13] Sovereign Wealth Fund Institute, (2015). Source on Sovereign Wealth Funds, Public Pensions, Superannuation Funds, Central Banks and Public Funds. [online] Available at: http://www.swfinstitute.org/fund-rankings/ [Accessed 10 Feb. 2015].
